# Reporting of Racial Health Disparities Research: Are We Making Progress?

**DOI:** 10.1200/JCO.21.01780

**Published:** 2021-10-25

**Authors:** Randy A. Vince, Nicholas W. Eyrich, Brandon A. Mahal, Kristian Stensland, Edward M. Schaeffer, Daniel E. Spratt

**Affiliations:** ^1^ Department of Urology, University of Michigan, Ann Arbor, MI; ^2^ Department of Radiation Oncology, University of Miami, Miami, FL; ^3^ Department of Urology, Northwestern University, Chicago, IL; ^4^ Department of Radiation Oncology, University Hospitals, Seidman Cancer Center, Case Western Reserve University, Cleveland, OH

Infiltrating virtually all aspects of scientific discourse is the persistent notion that race is a biologic construct. To this end, the use of race as a biologic variable to study health disparities may inadvertently promote a notion of biologic inferiority between races. But how did we arrive at this point? Specifically, within the United States, the concept of equality became a central pillar in developing the original 13 colonies; however, chattel slavery was a vital portion of the economic development. As such, this concept applied only to property-owning White men. With the rising anti-Black sentiment and prevailing thoughts that Whites were more intelligent and more human than Blacks, race was used to categorize groups of people based on physical characteristics and appearance.^1^ For this reason, the author Ta-Nehisi Coates brilliantly stated, “Race is the child of racism, not the father.”^2(p7)^

Several pseudoscientific theories, such as eugenics, were propagated in our society to assign biologic differences between races. The notion of biologizing race was prominent in medical training and research from our country's origin well into the 20th century. Although many have realized this is a false premise, the biomedical community continues to use race as a biologic variable. This practice occurs in medical education, research (academia, industry, and government), and the practice of race-based medicine (such as through clinical algorithms). Although the likely intent of race-based research and medicine is to use race as a surrogate of genetically inferred ancestry, the consequences of such practices are counterproductive to achieving equity and promotes the concept of racial essentialism.^1,3^ Regardless of the intent, the continuation of this practice promotes the notion that differences in health outcomes, specifically racial health disparities, are biologically based without the ability to modify the outcome.^4^ This is a central principle of racialization, whereby a dominant group ascribes a racial identity for purposes of continued social dominance, which is reinforced over time by society, particularly those in power.^5^

To properly interpret disparities data and target root causes, it is crucial to recognize that race is a social construct and racism is a sociopolitical tool used to promote White supremacy. In fact, to our knowledge, the frequency at which race and racism are described appropriately as socially driven entities in cancer research has yet to be reported. This information is vital if we seek to close the racial gaps in health care outcomes. Undoubtedly, education is a crucial component of combating the current issues we face as a profession and nation. Sharma et al^5^ describe one particularly dangerous example, noting that educators commonly discuss race without mentioning racism. To counteract the previously discussed practice, recent publications have provided guidance, precise definitions, and terminology of important concepts in disparities research that are often devoid from medical training (eg, race, structural racism, and social determinants of health).^6^ Although these efforts are essential, it is equally important to document how widespread the misuse of race is in racial health disparities literature and how frequently all forms of racism are acknowledged. Failing to recognize the prevalence of this practice in our research will continuously have downstream effects on our patients and society.

To understand the misuse of race in the scientific literature examining racial health disparities, we performed a systematic review of studies found after a MEDLINE (via PubMed) electronic search of manuscripts published between January 1960 and June 2020. All studies performed a comparative analysis of oncologic outcomes between physician or self-reported Black men to White or non-Black men with prostate cancer (Data Supplement, online only). Prostate cancer was selected as it represents one of the greatest racial disparities in oncology.^7^

**FIG 1.**
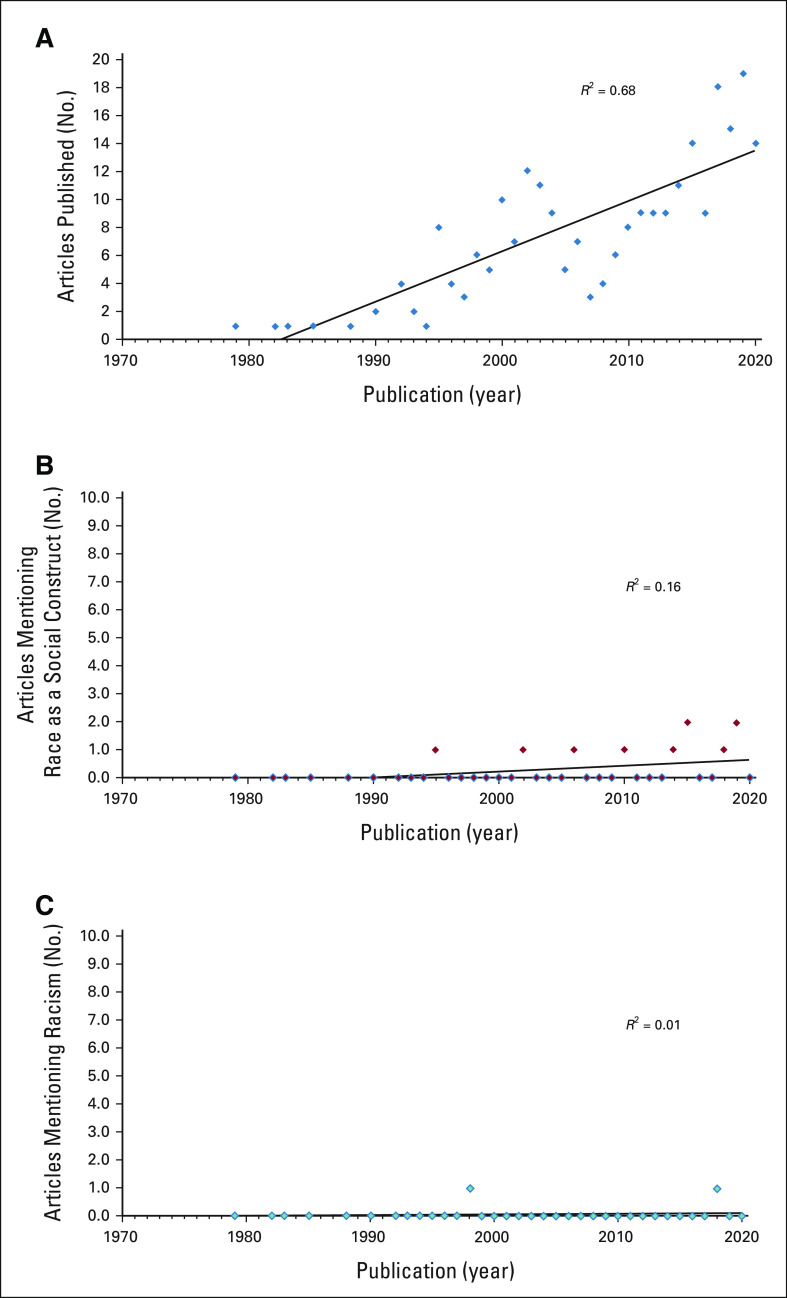
(A) Plot depicting the number of articles published annually performing comparative effectiveness research of prostate cancer outcomes by race. (B) Number of comparative effectiveness articles published annually that acknowledge race as a social construct. (C) Number of comparative effectiveness articles published annually that mention or acknowledge any form of racism. *R*^2^; square of the correlation coefficient.

A total of 249 studies met inclusion criteria. Only 4.0% (n = 10) acknowledged or interpreted race as a social construct, and 0.8% (n = 2) made any acknowledgment of racism (Fig 1). Perhaps just as alarming, we noted that although there is an increasing trend in the number of published articles examining racial disparities in prostate cancer over time (*R*^2^ = 0.68), there is weak to no correlation in the improvement of describing race as a social construct (*R*^2^ = 0.16) or the acknowledgment of racism over time (*R*^2^ = 0.01).

Thus, in 60 years, the acknowledgment of race and racism in the scientific literature does not appear to have changed. Our findings are consistent with the findings from Krieger et al,^8^ who also revealed that as a professional community, we have been negligent in the way we acknowledge race and racism.

Ultimately, these results propagate the notion that disparate prostate cancer outcomes are linked to African ancestry, despite the lack of data demonstrating an actual biologic driver to account for these differences. In a study of all publicly available sequencing data, Koga et al^9^ revealed that genomic alterations with clinical implications occur at similar rates between White and Black men. Moreover, in an analysis of gene expression data, PTEN loss, a genetic alteration with known associations with aggressive prostate cancer, occurred at lower rates in Black men.^10^ These studies refute any direct biologic basis for the disparities seen in prostate cancer. Yet, the dissemination of this long-held, high-problematic dogma continues while ignoring the impact of inequities, such as disparities in prostate cancer screening, treatment, and clinical trial enrollment as the culprit of differences in prostate cancer outcomes.^11-16^ This begs the question, if we closed the gap in health care access, would these differences in outcomes even exist?

Although the results of our analysis are specific to prostate cancer outcomes research, it would be unclear why one would assume these trends do not extend into other biomedical arenas.^17,18^ Likewise, an objective understanding of such trends represents a crucial first step in improving racial disparities research.

Although there is no method to quantify and assess the breadth and impact of racism fully, researchers need to acknowledge that the results of their work studying differences in health outcomes across different racial groups should be viewed through the lens of racism and other social determinants of health, rather than seeing race itself as the cause of disparate health outcomes. As initially discussed by Osborne and Feit,^19^ it is incredibly naïve to believe racial comparative research is performed in a vacuum and free of bias, as researchers operate as a part of a larger society that has for centuries upheld various forms of racism.

Therefore, it is imperative to recognize there's considerable overlap between structural racism and health. It has been extensively researched that the stress of racism and poverty leads to alterations in the HPA axis, causing increased cortisol levels and resulting in diabetes and abdominal obesity.^20^ Prolonged activation of this axis can result in cortisol resistance, thereby removing the anti-inflammatory effects of cortisol and stimulating chronic inflammation.^20^ Additionally, chronic stress because of interpersonal racism shortens telomere length, which is associated with chronic disease.^21^ These physiologic effects of racism overlapped with societal inequalities, such as reduced access to care, lead to disparate health outcomes.

Ultimately, the impact of structural and interpersonal racism leads to a vicious cycle of poverty, food insecurity, chronic stress, obesity, and decreased access to care, ultimately making racism the actual risk for differences in health outcomes, not race itself. Eliminating the use of race as a biologic variable is imperative, as for too long, this practice has prevented the biomedical community from doing the heavy lifting of addressing and overturning the structures, policies, and sentiments that truly lead to racial disparities.

There is a clear need to develop strategies to remedy this problem and bring about transformational change. Authors and journals are responsible for addressing the propagation of racialization throughout the scientific literature. We hope the biomedical community, like many other industries, is ready to not only change moving forward but also re-examine previously reported studies. If historical monuments, children's books (eg, Dr Seuss), and animated movies (eg, Disney) can take steps to remove or addend content with warnings of potential racial insensitivity, scientific journals should consider encouraging an erratum or warning on content that perpetuates the dangerous notion that race is a biologic construct and attributes direct health outcomes to race itself.

In closing, we again state that biomedical researchers and clinicians alike should eliminate the use of race as a biologic variable. Collectively, we call for our profession to pivot away from research attempting to identify differences between races to more extensively researching the unmitigated impact of various forms of racism to push forward policy change with an ultimate goal of equity. We recognize that the policies and actions that lead to these disparities have been enacted over centuries. But, without intentional acknowledgment, reconciliation and purposeful investment progress will continue to be stymied. Our profession has a moral responsibility to use all available means to bring about such transformative change.
